# Orthopaedic cardiac considerations in emergency

**DOI:** 10.1051/sicotj/2021051

**Published:** 2021-11-05

**Authors:** Spyridon Katsanos, Theodosis Saranteas, Andreas F. Mavrogenis

**Affiliations:** 1 Department of Emergency Medicine and Cardiology, National and Kapodistrian University of Athens 11527 Athens Greece; 2 Second Department of Anesthesiology, National and Kapodistrian University of Athens 11527 Athens Greece; 3 First Department of Orthopaedics, National and Kapodistrian University of Athens, School of Medicine 11527 Athens Greece

**Keywords:** Cardiology, Anesthesia, Echocardiogram, Ultrasonography, Aortic stenosis, Orthopaedic surgery, Emergency

## Abstract

Orthopaedic patients undergoing emergency orthopaedic surgery should be referred for cardiac evaluation only when they are symptomatic or when a specific cardiac intervention is expected to reduce the surgical risk. A preoperative delay of 24–48 h of emergency orthopaedic operations has been associated with increased mortality and poor functional status of the patients. Research in the preoperative setting is almost exclusively retrospective because randomized studies are difficult to be performed and pose serious ethical concerns. Moreover, inevitably, guidelines have a low level of evidence and do not always provide a straightforward framework for the preoperative care of the patients. This editorial revisits the most common clinical cardiology dilemmas for emergency orthopaedic surgery to explore controversies of current recommendations and elaborate on the role of echocardiography in the perioperative period in emergency orthopaedic surgery.

Orthopaedic patients undergoing emergency orthopaedic surgery should be referred for cardiac evaluation only when they are symptomatic or when a specific cardiac intervention is expected to reduce the surgical risk [1–4]. In the preoperative evaluation, a time delay for echocardiography (18–35%) and/or pharmacological stress tests (7%) are not uncommon [5–7]. Interestingly, a delay of 24–48 h of emergency orthopaedic operations has been associated with increased mortality and poor functional status of the patients [1, 2]. Therefore, adherence to preoperative guidelines for a non-cardiac surgery, although frequent, it is controversial whether implementation of guidelines may improve the prognosis [2, 8, 9]. Research in the preoperative setting is almost exclusively retrospective because randomized studies are difficult to be performed and pose serious ethical concerns [3, 4]. Moreover, inevitably guidelines have a low level of evidence and do not always provide a straightforward framework for the preoperative care of orthopaedic patients.

Although orthopaedics requires a wide spectrum of technical skills and theoretical knowledge, very important in modern times to collaborate with other medical specialities such as cardiologists, anesthesiologists, diagnostic and interventional radiologists, oncologists, and medical pathologists, and endocrinologists/metabolic bone disease specialists is inevitable. To emphasize the orthopaedics collaboration between these specialities, we aim to publish an editorial series to acknowledge what orthopaedics can obtain from related specialities and to consolidate in their practice to provide the best care for orthopaedic patients. In this context, the present article reviews the most common clinical cardiology dilemmas for emergency orthopaedic surgery to explore controversies of current recommendations and to elaborate on the role of echocardiography in the perioperative period in emergency orthopaedics.

## Echocardiography at baseline evaluation

A thorough medical history and complete physical examination are necessary for all orthopaedic patients referred for cardiac clearance preoperatively. The definition of the functional capacity of the patient is the cornerstone of preoperative care. Patients who can walk up two flights of stairs or walk up a hill practically do not need any further testing [3, 4]. As part of the basic workup of the patients, the Lee index should be calculated. Patients who undergo low-risk orthopaedic surgery do not need electrocardiography evaluation according to guidelines; however, echocardiograms are easy, widely available, and give critical information. In previous decades, heart auscultation was the standard to determine heart valve pathology; nonetheless, currently, this skill is gradually deteriorating [10]. For example, even expert cardiologists may miss aortic valve severity in 30% of cases, whereas a combined aortic and mitral valve pathology may be missed 45% of the time [11].

According to current guidelines, echocardiography is limited to patients with newly dialogued or newly worsened dyspnea and steady patients with ≥moderate valvular stenosis or regurgitation, without a prior echocardiography study in the same year [3, 4]. However, only 25% of anesthesiologists are confident to proceed with the surgical operation of orthopaedic patients with cardiac murmurs based only on their clinical examination, whereas 20% of them will insist upon an echocardiogram to be performed irrespectively to the patients’ symptoms [12]. Non-guidelines indicated echocardiography is practically the norm for orthopaedic surgery, as shown in various single centre and national-wide studies [2, 8, 12, 13]. In almost all cases, the referral for echocardiography is related to the time delay of the operation, and definitely, it does not improve the outcomes. However, these studies may have a contrary view: patients undergoing preoperative echocardiography are more commonly nonagenarians and have a medical history of heart failure, previous myocardial infarction, valvular heart disease, chronic renal failure, diabetes mellitus, dementia, etc. [2, 8, 12, 13]. This is genuinely a group of frail patients whose functional capacity cannot be evaluated, and their medical history is often poorly illustrated. They also suffer from cardiac and non-cardiac conditions that influence their inhospitable mortality [14]. Additional information for this group of orthopaedic patients is valuable and may alter their medical and anesthesiological management [2, 8, 13]. No doubt, the cost remains a major concern for off-label echocardiography, especially when resources are stretched.

Bedside, portable, point of care echocardiography (POCE) may be an excellent fast alternative to standard echocardiography. This technique guarantees the quality of care at a lower cost. On top of physical examination, POCE can dramatically step up diagnostic sufficiency of medical students and junior doctors with no previous experience in echocardiography [15]; after only 2–4 h of training, their view of the left ventricular function and major valvular abnormalities matches that of expert cardiologists [15]. Randomized studies have shown that POCE is not inferior to standard echocardiography for patients undergoing non-cardiac surgery [16]. Additionally, POCE is an invaluable clinical tool in the busy and noisy emergency setting where cardiac auscultation can be problematic. Certainly, there are issues regarding training and competency; however, acquired by accredited junior doctors, POCE can be a trustworthy replacement of a standard echocardiogram and a gatekeeper to unnecessary cardiac consultation. In our opinion, this is an example where breakthrough innovation has outpaced guidelines recommendations.

## Non-invasive stress tests

Myocardial perfusion scintigraphy and stress echocardiography are commonly recommended for orthopaedic patients undergoing emergency surgery, but they rarely lead to cardiac catheterization [5, 6]. Coronary computed tomography angiography is not a first-line examination for elderly patients, and magnetic resonance imaging and positron emission tomography of the heart are not widely available. Preoperative stress-testing in emergency orthopaedic surgery may be performed in patients with questionable cardiac symptoms, a history of known coronary artery disease, as well as those with a high-risk profile and poor (≤4 metabolic equivalents) functional status. Non-invasive stress testing as a guideline is recommended only in subgroups of patents patients undergoing elective orthopaedic surgery [1, 2]. This controversial use of non-invasive stress tests may be explained as a more time-flexible approach to non-life saving surgery that could be postponed under specific circumstances.

A negative stress test will be welcome by the surgical team treating the patient; however, a positive stress test commonly leads to an impasse. The team usually takes time to interpret results and contemplate their options. In this phase, there may be considerable opinion differences. When cardiologists are confronted with clinical and imaging data of patients scheduled for non-cardiac surgery, there is a likelihood of 54% for discordance in recommendations for prophylactic revascularization with a 26% chance to be contradictory [17]. Most importantly, stent implantation will ensure dual antiplatelet therapy and at least a three months time delay of the surgical operation [1, 2]. Additionally, patients may require weeks to recover from coronary artery bypass graft surgery. Of course, before assigning all the blame for over-testing to the cardiologists, we should acknowledge that defensive medicine is an indigenous problem to all modern healthcare systems [18]. Simply put, cardiologists are aware of their aggressive practice, however, they feel that in a likely malpractice sue, they will defend their case better by ordering an extra test rather by explaining why they did not order it [18]. From a surgeon’s point of view, defensive medicine would be to avoid performing such a hazardous but necessary procedure. The solution to the overuse of stress tests is not easy. Sometimes, overuse of imaging is a substitute for insufficient personal contact, and in this perspective financial incentives could help [18].

In everyday practice, most clinical dilemmas arise when there is an ad hoc diagnosis of coronary artery disease during the preoperative cardiac evaluation. It is not uncommon that ischemic resting electrocardiographic findings such as Q-waves, ST-segment depression and T-inversions, and left ventricular wall motion abnormalities are first diagnosed during the preoperative cardiac evaluation of patients undergoing emergency surgery. These otherwise clinically steady patients fall into the category of chronic coronary syndromes according to the 2019 guidelines of the European Society of Cardiology (ESC) [19]. Still, non-invasive testing of the patients before the orthopaedic surgery does not make much sense for risk stratification if it cannot alter management. The problem is that applicable ESC recommendations for stable coronary artery disease patients leave a revascularization option open, such as for patients with a large (>10%) left ventricular area of ischemia, >90% coronary artery stenosis, and ≤35% left ventricular ejection fraction due to coronary artery disease [19]. It is reasonable that this ischemic profile may be associated with a high perioperative risk, but on the other hand, prophylactic revascularization is not supported by related published studies [20, 21].

We should admit that stress testing is reasonable only if revascularization is an option. In contrast, any revascularization strategy will be associated with a considerable time delay of the emergency surgery. Instead, patients and treating physicians should be confronted upfront with this elementary dilemma and avoid redundant delays, and guideline providers should acknowledge that over-testing of the patients in urgent orthopaedic surgery is the mainstream practice [5, 6]. Future guidelines should provide a straightforward framework for the fast-track preoperative management of this group of patients.

## The paradigm of aortic stenosis

Degenerative valvular disease increases dramatically with age in industrialized countries. Aortic stenosis may be present in 4.6% of patients aged >75 years, while aortic regurgitation (1.7%) and mitral stenosis (0.2%) are less common [7]. In clinical practice, known or suspected aortic stenosis is a common reason for cardiac consultation before surgery. Aortic stenosis is usually defined by aortic valve area ≤1 cm^2^, peak systolic flow velocity ≥4 m/s, or mean gradient ≥40 mmHg, with significant deviations among patients groups [22–27]. Aortic stenosis has been related to the worst perioperative outcome in most studies, including intermediate and high-risk surgery [22–27]. There is commonly an increase in the combined endpoint of mortality, perioperative heart failure, and myocardial infarction, which is not confirmed in all studies [22–27]. These complications result from perioperative oxygen supply-demand imbalances due to hypotension, tachycardia, and concomitant impaired coronary perfusion [25]. Few studies have focused on the entity in orthopaedic patients, and their results have been inconsistent [22–24]. The discrepancies have been attributed to the different design of the studies, the improvement of anesthesiology management overtime, but also to heterogeneous definitions of endpoints, including troponin elevation with or without electrocardiographic changes, cardiac death vs. any death, regular monitoring of all patients vs. symptom-based monitoring, and more [25–27].

In the emergency setting, current guidelines support valvuloplasty only for symptomatic severe aortic stenosis [1, 2]. This actually leaves room for different interpretation of our echocardiography findings that current guidelines could support. In fact, medical history is ill-defined in frail patients, and cardiac symptoms are difficult to distinguish under the influence of analgesia, acute pain, and psychological distress. Also, any time delay for intervention could be justified on the ground of clearly improved outcomes; yet, to date, only a few retrospective data support this approach [28]. Additionally, valvular intervention is not without consequences: valvular replacement may be of unexpectable cardiac risk, balloon aortic valvuloplasty has been linked to stroke, coronary ostia occlusion and acute renal failure, whereas transcatheter aortic valve implantation requires time-consuming pre-procedural work-up and dual antiplatelet therapy for 3–6 months. In real-world data, there is a significant pool of patients that proceed with non-cardiac surgery despite severe aortic stenosis. This is because they usually undergo emergency surgery, have prohibitive surgical risk or simply because they deny open-heart surgery [26, 27].

Novel echocardiography techniques and multimodality imaging has revolutionized the risk stratification of patients with aortic stenosis [29]. This information is very relative for the hypovolemic trauma patient. For example, the aortic gradient is more flow-dependent than aortic valve area. We already know that a higher gradient of the aortic valve may be related to increased mortality peri-orthopaedic surgery [30]. The introduction of stoke volume, global strain and novel markers of diastolic dysfunction in standard preoperative evaluation of severe aortic stenosis could give a better risk stratification and define patients who will truly benefit from prophylactic aortic valve surgery/intervention [29]. Likewise, orthopaedic patients with mild aortic stenosis that would otherwise get cardiac clearance for surgery may prove to be, in fact, of very high risk in the presence of heart failure [31].

## Perioperative echocardiographic monitoring

In orthopaedic patients, transthoracic (TTE) and transesophageal (TEE) echocardiography are valuable diagnostic tools that can be used interchangeably in conjunction with other modalities such as the electrocardiogram and computed tomography (CT) for the diagnosis of cardiovascular complications and emergency conditions in the perioperative period [32]. In patients undergoing orthopaedic surgery, although with certain limitations [32, 33], echocardiography can be used successfully to assess and monitor cardiovascular hemodynamics by examining the left ventricular function and the cardiac preload through measurements of the heart chambers and inferior vena cava (IVC) diameters [32].

Bone cement implantation syndrome (BCIS) is an intraoperative complication in patients receiving different types of cemented prostheses. Several mechanisms have been put forward, mainly involving the release of methyl methacrylate (MMA) cement monomer into the circulation after cementation and the release of other sources of emboli such as air, bone marrow, fatty particles, cement molecules, and fibrin aggregations. BCIS can present as transient desaturation, hypotension, cardiac dysrhythmias and cardiovascular collapse, leading to death in 0.6–1% of the patients [34]. Once hemodynamic perturbations are present during cement implantation, echocardiography, either TTE or TEE, may be routinely employed to assess cardiac function. On these occasions, the intraoperative use of echocardiography yields sufficient information about the cardiovascular hemodynamics, and importantly can help with the diagnosis and management of BCIS-related hypotension. The thrombotic materials and massive embolic showers, which pass through the circulation into the right heart chambers, can be explicitly visualized, and their fallouts on heart function can be easily diagnosed [34]. The echocardiographic findings of BCIS encompass acute right ventricular (RV) dilation, regional right ventricle akinesia of mid-free wall segment with normal motion at the apex (McConnell’s sign), septal flattening, compressed left ventricle, and inferior vena cava dilatation. Moreover, in time-critical situations such as cardiac arrest, prompt diagnosis of the underlying cause can be of fundamental importance; in these cases, not only does echocardiography play a significant role in the diagnosis of cardiac arrest, but by guiding cardiopulmonary resuscitation (CPR) efforts, it can assist with the evaluation of cardiac function during CPR [35, 36].

The cardiovascular system also sustains certain alterations during the ageing process, with dehydration being a very common condition among elderly patients [37]. In geriatric orthopaedic patients, spinal anaesthesia induces severe intraoperative hypotension leading to postoperative complications necessitating intensive and prolonged resuscitative efforts [38]. TTE is an important perioperative monitoring tool for assessing left ventricular function and intravascular volume status (through assessment of the cardiac chambers and IVC diameters) [39, 40]. In addition, preoperative assessment of the maximum diameter of the IVC during expiration (dIVCmax), the IVC collapsibility during the respiratory cycle as well as the (dIVCmax) -to- IVC collapsibility index ratio can predict spinal-induced hypotension events in elderly patients undergoing orthopaedic surgery [41].

## Legal issues

Documentation of the patient’s assessment is of great importance for any future negligence claims [42]. The medical examination should exclude active conditions that would postpone surgery, such as unstable angina, acute or recent (≤30 days) myocardial infarction, acute heart failure, symptomatic valvular disease, and significant arrhythmias (high-grade atrioventricular block/ventricular arrhythmias). Medical records should include all components of the Lee index such as the history of coronary artery disease, heart failure, cerebrovascular disease, insulin-dependent diabetes mellitus and serum creatinine >2 mg/dL [42]. The risks of the procedure should be explained with disclosure of their exact frequency. A Lee index of 1 is associated with a 0.9% risk of major cardiac events, whereas an index of ≥3 with a 11% risk. Correspondingly, the likely outcomes with no treatment should be explained. Cardiologists can record their rationale for medical thinking. The physician is expected to provide the best options based on up-to-date evidence that would align with what an average physician with the same skills would do in an analogous situation. However, serious concerns arise in emergency surgery where patients are too ill to comprehend the risks and communicate their choices. In real life, up to 68% of patients undergoing hip arthroplasty do not recall medical information given during their consent interview [43]. Physicians are encouraged to inform and conform themselves to local laws and regulations about legal incapacity. A daedal law system makes physicians more hesitant to give their stamp of approval for immediate surgery.

In conclusion, written consent must be obtained by all patients or their relatives after a thorough analysis of the procedure and the potential cardiac risks and complications. The medical team should be accountable to acknowledge the risks stemming from the medical history of the patient relative to the severity of the orthopaedic surgery [40–48]. Well trained and experienced cardiologists, anesthesiologists and orthopaedic surgeons should harmonically cooperate and undertake successfully all the difficult cases. Exhaustive and scrupulous preoperative cardiovascular examination is of fundamental importance. Individualized, well fitted with the severity of the cardiac pathology, anaesthesia techniques, alone or in combination (peripheral, central nerve blocks, general anaesthesia techniques) must always be implemented. Meticulous perioperative monitoring, including echocardiography, in addition to high standards and quality of orthopaedic care, are imperative and the cornerstone of our practice.

## Conflicts of interest

The authors declare that they have no relevant financial or non-financial interests to report. Author AF Mavrogenis is co-Editor-in-Chief of SICOT-J.

## Funding

This research did not receive any specific funding.

## Ethical approval

Ethical approval was not required.

## Informed consent

This article does not contain any studies involving human subjects.

## Author contributions

S. Katsanos: Writing original draft, Investigation; Visualization; T. Saranteas: Conceptualization, Methodology, Visualization; A.F. Mavrogenis: Supervision, Writing, Reviewing and Editing.
